# Biotic and Abiotic Stresses Activate Different Ca^2+^ Permeable Channels in *Arabidopsis*

**DOI:** 10.3389/fpls.2017.00083

**Published:** 2017-01-31

**Authors:** Xiao-Qiang Cao, Zhong-Hao Jiang, Yan-Yan Yi, Yi Yang, Li-Ping Ke, Zhen-Ming Pei, Shan Zhu

**Affiliations:** ^1^College of Life and Environmental Sciences, Hangzhou Normal UniversityHangzhou, China; ^2^Department of Biology, Duke University, DurhamNC, USA; ^3^Key Laboratory of Plant Secondary Metabolism and Regulation of Zhejiang Province, College of Life Sciences, Zhejiang Sci-Tech UniversityHangzhou, China

**Keywords:** biotic stress, abiotic stress, flg22, Pep1, aequorin-based Ca^2+^ imaging, calcium signal

## Abstract

To survive, plants must respond rapidly and effectively to various stress factors, including biotic and abiotic stresses. Salinity stress triggers the increase of cytosolic free Ca^2+^ concentration ([Ca^2+^]_i_) via Ca^2+^ influx across the plasma membrane, as well as bacterial flg22 and plant endogenous peptide Pep1. However, the interaction between abiotic stress-induced [Ca^2+^]_i_ increases and biotic stress-induced [Ca^2+^]_i_ increases is still not clear. Employing an aequorin-based Ca^2+^ imaging assay, in this work, we investigated the [Ca^2+^]_i_ changes in response to flg22, Pep1, and NaCl treatments in *Arabidopsis thaliana*. We observed an additive effect on the [Ca^2+^]_i_ increase which induced by flg22, Pep1, and NaCl. Our results indicate that biotic and abiotic stresses may activate different Ca^2+^ permeable channels. Further, calcium signal induced by biotic and abiotic stresses was independent in terms of spatial and temporal patterning.

## Introduction

In the natural environment, plants have to continuously cope with various stress factors, such as salt, drought, attacks of herbivorous insects, and invasion of microbial pathogens. To survive, plants should respond rapidly and effectively to each stressor. Recent studies revealed that about 10 million hectares of agricultural land are abandoned every year due to high salinity (Zhu, 2001, 2003; Munns and Tester, 2008). Plant diseases cause massive losses in agricultural yields as well as abiotic stresses (Singh et al., 2011; Dangl et al., 2013; Yoshida et al., 2013). Moreover, the simultaneous occurrence of different stresses results in a high degree of complexity in terms of plant responses, as the responses to the combined stresses are largely controlled by different, and sometimes opposing, signaling pathways that may interact and inhibit each other (Suzuki et al., 2014). Therefore, it is critical to study how plants respond to both biotic and abiotic stresses.

The calcium, which serves as a secondary messenger, is thought to be a key element in plants to understand how a sophisticated network of signaling pathways respond to various abiotic and biotic stimuli (Hetherington and Brownlee, 2004; Pandey et al., 2004; Dodd et al., 2010; Yuan et al., 2014). The calcium (Ca^2+^) signaling has been implicated in regulating many perspectives of plant growth and responses to the environment (McAinsh and Pittman, 2009; Dodd et al., 2010; Kudla et al., 2010). Immunity of plant activates two signal transduction pathways, i.e., Ca^2+^ signaling pathways and cytoplasmic mitogen-activated protein kinase, thus leading to transcriptional reprogramming and accumulation of chloroplast-derived reactive oxygen species (ROS; Zhang et al., 2007; Boudsocq et al., 2010; Dubiella et al., 2013; Li et al., 2014) as well as generation of defense-related hormones, e.g., jasmonic acid and salicylic acid (Grant and Jones, 2009; Klauser et al., 2015). Abiotic stress also triggers a calcium-signaling cascade in plants, leading to transcriptional regulation and subsequent physiological as well as developmental responses. Salt stress is a representative of such abiotic stresses. Although the molecular mechanisms surrounding the initial perception of salt stress are unknown, it is now well established that salt stress triggers a transient increase in cytosolic Ca^2+^ concentration ([Ca^2+^]_i_) that lasts for approximately 2 min (Knight et al., 1997; Tracy et al., 2008; Jiang et al., 2013).

These specific Ca^2+^ signatures are formed as a result of the tightly regulated activities of Ca^2+^ channels and transporters in different tissues, organelles, and membranes (Rentel and Knight, 2004; Kudla et al., 2010; Spalding and Harper, 2011; Batistic and Kudla, 2012; Stael et al., 2012). In terms of plant immunity, the changes of [Ca^2+^]_i_ are detected by cytosolic Ca^2+^ sensors. One of the earliest signaling events following the perception of microbe-associated molecular patterns (MAMPs) or damage-associated molecular patterns (DAMPs) is a rapid change of [Ca^2+^]_i_ and concomitant membrane depolarization (Blume et al., 2000; Lecourieux et al., 2002; Ranf et al., 2008; Jeworutzki et al., 2010; Nomura et al., 2012; Li et al., 2014). Consequently, the generation of ROS could restrict the growth of pathogen via cell wall strengthening and toxic effects, or initiate signaling functions (Torres et al., 2002; Chinchilla et al., 2007; Ranf et al., 2011; Kadota et al., 2015). As the first line of innate immunity, pattern-recognition receptors (PRRs) can recognize MAMPs in the plasma membrane and trigger a series of basal defense responses (Macho and Zipfel, 2014; Yamada et al., 2016). Plant nucleotide-binding and leucine-rich repeat (NB-LRR) proteins, encoded by plant “R” genes, recognize pathogen-derived effector proteins and trigger hypersensitive response (Tsuda and Katagiri, 2010; Zipfel, 2014). The well-studied pathogen-associated molecular pattern (PAMP)/PRR pairs in *Arabidopsis* so far are EF-Tu/EFR (elongation factor thermo unstable receptor) and flagellin/FLS2 (flagellin-sensitive 2), with the peptides elf18 and flg22, respectively, functioning as the elicitor-active PAMPs (Felix et al., 1999; Gomez-Gomez et al., 1999; Gomez-Gomez and Boller, 2000; Kunze et al., 2004; Zipfel et al., 2006). Other biotic stresses, as well as Pep1, a plant-derived DAMP, have been reported in recent years (Huffaker et al., 2006; Huffaker and Ryan, 2007; Shan et al., 2008; Krol et al., 2010).

From recent studies, we know that both biotic and abiotic stresses can trigger a rapid increase in cytosolic Ca^2+^. Jiang et al. (2013) reported that NaCl-gated Ca^2+^ channels and H_2_O_2_-gated Ca^2+^ channels may be differ. This study also suggests that NaCl- and H_2_O_2_-evoked [Ca^2+^]_i_ may reduce the potency of both NaCl and H_2_O_2_ in triggering [Ca^2+^]_i_ increases, highlighting the existence of a feedback mechanism. Alternatively, NaCl and H_2_O_2_ may activate the same Ca^2+^ permeable channel, which is expressed in different types of cells and/or activated via different signaling pathways. However, it is still not clear whether biotic and abiotic stress-activated Ca^2+^ channels influence each other or they are independent of each other. Moreover, the activation of Ca^2+^ channels by different biotic stresses (e.g., MAMP/DAMP) is a topic, which is also worthy of investigation.

In this study, we systematically investigated and analyzed the relationship and interaction between biotic and abiotic stresses in *Arabidopsis*. We found that the increases of [Ca^2+^]_i_ induced by both stimuli were higher than those induced by a single stress, suggesting that biotic and abiotic stresses have an additive effect on [Ca^2+^]_i_. We also found that flg22-induced [Ca^2+^]_i_ increases may inhibit both PAMP- and DAMP-activated [Ca^2+^]_i_ channels via a feedback mechanism, but not abiotic-activated [Ca^2+^]_i_ channels. These results suggest that the responses involve in both inhibitory feedback mechanisms, as well as an interaction between the stimuli-mediated Ca^2+^ signaling pathways.

## Materials and Methods

### Plant Materials and Growth Conditions

*Arabidopsis thaliana* ecotype Col-0 constitutively expressing intracellular Ca^2+^ indicator aequorin (pMAQ2) is a gift from M. Knight and the principles of how the active aequorin is formed can be found in Knight et al. (1991). *Arabidopsis* plants were grown in 150 mm × 15 mm round Petri dishes in half-strength Murashige and Skoog salts (MS; Gibco), supplemented with 1.5% (w/v) sucrose (Sigma), and 0.8% (w/v) agar (Becton Dickinson) adjusted to pH 6.0 with KOH in controlled an environmental room at 21 ± 2°C. The fluency rate of white light was ∼110 μmol m^-2^ s^-1^. The photoperiods were 16 h light/8 h dark cycles. Seeds were sterilized with 2.5% plant preservative mixture (Caisson Laboratories) and stratified at 4°C for 3 days in the dark, and then transferred to the growth room.

### Aequorin Reconstitution and Measurement of [Ca^2+^]_i_

*Arabidopsis thaliana* plants expressing cytosolic apoaequorin were used for [Ca^2+^]_i_ measurements (Knight et al., 1991; Tang et al., 2007). Sixty-four seedlings were grown on half-strength MS medium for 8 days. Reconstitution of aequorin was performed *in vivo* by spraying seedlings with 3.3 mL of 10 μM coelenterazine (from Prolume) per Petri dish followed by incubation at 22°C in the dark for 8 h. Treatments and aequorin luminescence imaging were performed at room temperature using a ChemiPro HT system, which includes a cryogenically cooled and back-illuminated charge-coupled device (CCD) camera, liquid nitrogen autofiller, camera controller, and computer-equipped WinView/32 software (Roper Scientific) as described previously (Tang et al., 2007). The CCD camera has a 1300 × 1340 pixel resolution and is cooled to -120°C by the cryogenic cooler system prior to image recording. The recording was started 80 s prior treatments and luminescence images were taken every 20 s or continuous 7 min. The total remaining aequorin was estimated by treating plants with a discharging solution containing 0.9 M CaCl_2_ in 10% (v/v) ethanol and recorded for 5 min until values were within 1% of the highest discharge value (Tang et al., 2007; Ranf et al., 2012; Yuan et al., 2014). The recorded luminescence images were analyzed using Meta Morph 7.7 and WinView/32. Here, the Ca^2+^ level depicted as *L*/*L*_max_ ratio correlates with the light emission from aequorin. To calculate the ratio, the actual aequorin luminescence, denoted as *L*, at any sampling point is normalized by the total remaining aequorin (Knight et al., 1996; Ranf et al., 2012). The experiments were carried out under room temperature between 22 and 24°C.

### Elicitors and NaCl Treatments

For stress treatments, Petri dishes were placed individually into the ChemiPro HT chamber and luminescence images were started 80 s prior the treatment and taken at 20 s intervals or 7 min continuously. The treatment solution (100 mL) at 1 μM concentrations of flg22 and Pep1 (Felix et al., 1999; Huffaker et al., 2006), which were synthesized by China Peptide^1^ or 200 mM NaCl (Sigma) was added into Petri dish in the dark, and luminescence was recorded. For changes in bath solution, a four-channel peristaltic pump (Dynamax RP-1, Rainin) was used to perfuse Petri dish with water as indicated in the figures. Then, additional stress treatment was applied by adding 100 mL solution into Petri dish.

## Results

### Dose-Dependence and Kinetics of flg22- and Pep1-Induced [Ca^2+^]_i_ Increases

Changes in cytosolic Ca^2+^ concentration ([Ca^2+^]_i_) can be monitored by the bioluminescent Ca^2+^-binding protein aequorin *in vivo* (Knight et al., 1991). Apoaequorin can be expressed in plants and spontaneously reconstitutes to functional holo-aequorin upon addition of the native luminophore coelenterazine (CTZ-n) or chemically modified derivatives, such as coelenterazine-h (CTZ-h), for enhanced sensitivity (Shimomura et al., 1993; Mithofer and Mazars, 2002).

**FIGURE 1:**
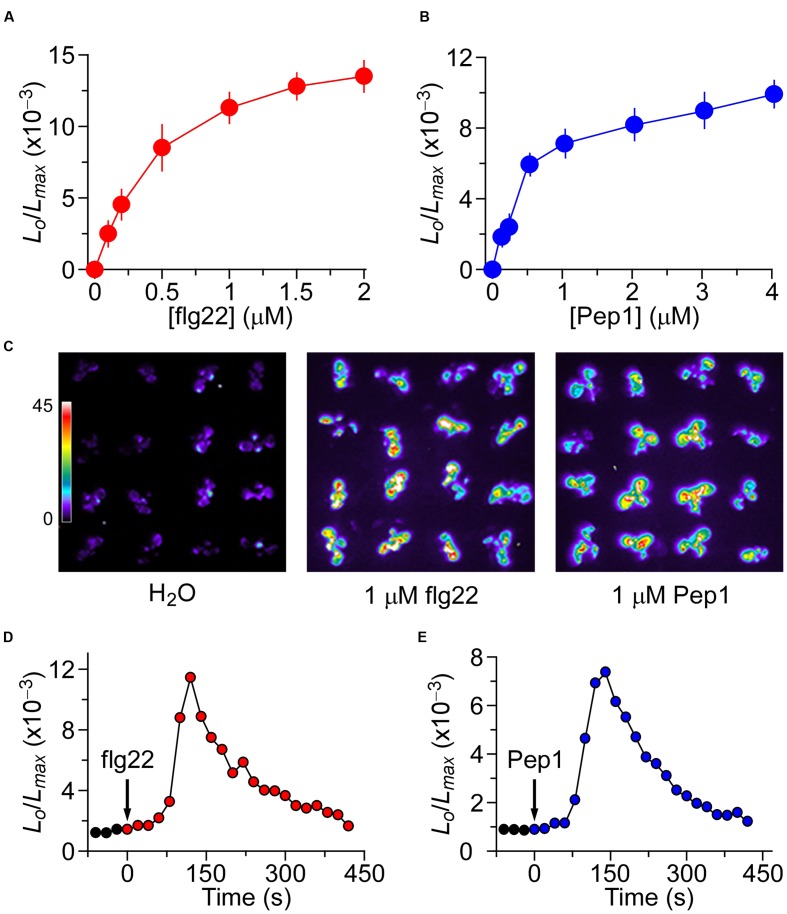
**Increases in [Ca^2+^]_i_ in response to flg22 and Pep1 treatments.** **(A,B)** Increases in [Ca^2+^]_i_ induced by several concentrations of flg22 **(A)** and Pep1 **(B)** in *Arabidopsis*. Seedlings expressing aequorin and grown for 7 days were treated with solutions containing several concentrations of flg22 or Pep1, and aequorin images were taken every 20 s for 420 s, and the peak value was recorded. Data for four independent experiments are shown (mean ± SEM; *n* = 16). **(C)** Imaging of [Ca^2+^]_i_ increases in response to the treatments of H_2_O, 1 μM flg22 and 1 μM Pep1. Pictures were taken for 420 s. **(D,E)** Time courses of increases in [Ca^2+^]_i_ induced by 1 μM flg22 **(D)** or 1 μM Pep1 **(E)**. Seedlings grown for 7 days were treated with flg22 and Pep1 at time 0, and aequorin images were taken every 20 s. Similar results could also been observed in four independent experiments using 256 seedlings.

To determine whether the mechanisms behind the increases of [Ca^2+^]_i_ induced by biotic and abiotic stresses are interrelated in *Arabidopsis*, we first attempted to identify the optimum concentrations of flg22 and Pep1 that ideally could be applied to generate about half of the maximum amplitude of [Ca^2+^]_i_ required for potential up- and down-regulation. Furthermore, we attempted to establish the kinetics of flg22- and Pep1-induced [Ca^2+^]_i_ increases so as to administer these stresses in different sequential combinations. To analyze flg22-induced increases in [Ca^2+^]_i_, we treated *Arabidopsis* seedlings expressing aequorin with solutions containing 0 to 2 μM flg22. Aequorin bioluminescence images were recorded every 20 s for 600 s. The Ca^2+^ level correlates with the light emission from aequorin and is depicted as the ratio of *L*/*L*_max_, where the actual aequorin luminescence (*L*) at any measurement point is normalized to the total remaining aequorin (Knight et al., 1996). Plants grown on the half-strength MS medium had an average basal [Ca^2+^]_i_ of 80 ± 21 nM (Dodd et al., 2010). As expected, the [Ca^2+^]_i_ increased in response to flg22 treatment (**Figure 1A**). The magnitudes of [Ca^2+^]_i_ increases were found to be dependent on the concentration of flg22, with a higher concentration of flg22 evoking a greater increase in [Ca^2+^]_i_. The flg22 concentration required for a half-maximal response (i.e., the half of elicitor concentration which induced maximal [Ca^2+^]_i_ increase) was 1 μM, which was chosen as an optimum concentration for the subsequent analysis of interaction with Pep1- and NaCl-induced increases in [Ca^2+^]_i_. Next, we determined the temporal dynamics of flg22-induced [Ca^2+^]_i_ increases under the imposed experimental conditions as a control for further comparison (**Figure 1D**). We found that [Ca^2+^]_i_ increased immediately after the application of 1 μM flg22, reached a peak at about 120 s, and then declined gradually (**Figure 1D**). Imaging aequorin bioluminescence for less than 20 s resulted in images with a low signal-noise ratio in our system. Thus, the temporal resolution was set at about 20 s, which was sufficient for the current study. At about 200 s, the [Ca^2+^]_i_ was reduced to a new resting level. Similarly, we analyzed the increases in [Ca^2+^]_i_ in response to Pep1. Seedlings were treated with different concentrations of Pep1 from 0 to 4 μM, and [Ca^2+^]_i_ was analyzed. As expected, Pep1 induced increases in [Ca^2+^]_i_ in a dose-dependent manner (**Figure 1B**). The [Ca^2+^]_i_ increases recorded after single treatments were consistent with the results described above (**Figure 1C**). The Pep1 concentration required to achieve a half-maximal response was around 1 μM, with the magnitude of [Ca^2+^]_i_ similar to that induced by 1 μM flg22. We then determined the temporal dynamics of the [Ca^2+^]_i_ increase induced by 1 μM Pep1. Following treatment with 1 μM Pep1, the [Ca^2+^]_i_ increased and reached a peak at 140 s (**Figure 1E**), then it took another 250 s for the [Ca^2+^]_i_ to reach a new basal level. In overall, it seems that the increases of [Ca^2+^]_i_ occur faster in response to flg22 than Pep1, but reset to a resting level 400 s after the treatment.

### The Crosstalk between flg22- and Pep1-Induced [Ca^2+^]_i_ Increases

**FIGURE 2:**
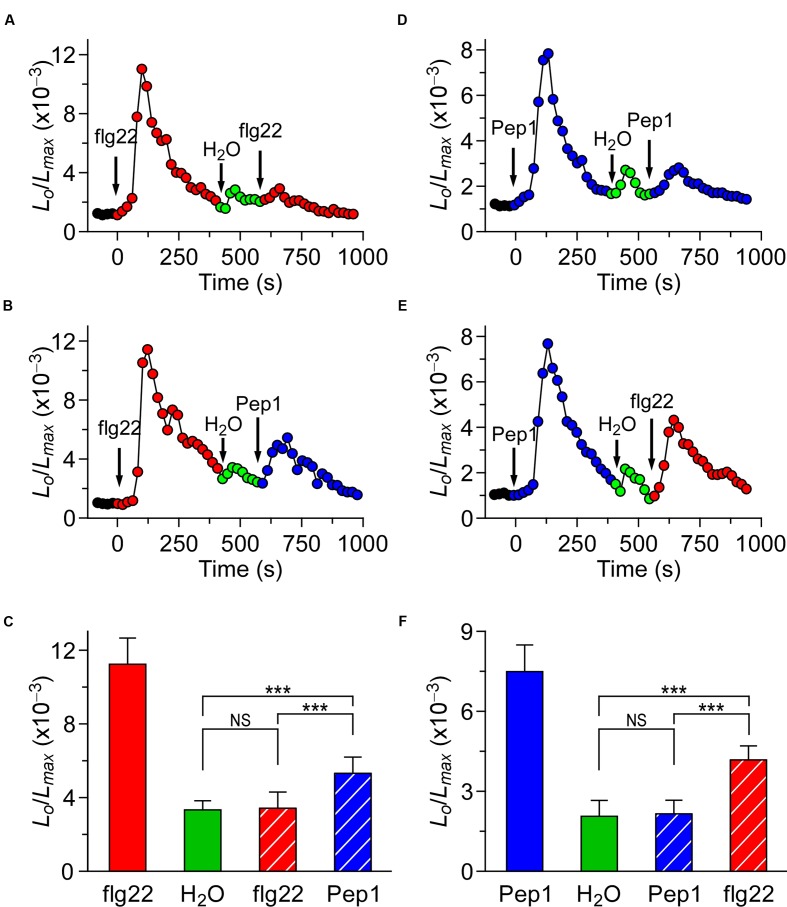
**flg22 and Pep1 induced [Ca^2+^]_i_ increases partly influence each other.** **(A,B)** *Arabidopsis* seedlings were subjected to a 1 μM flg22 treatment once at 0 s, and the solution was perfused by deionized water at 420 s. Then, a second 1 μM flg22 **(A)**, or 1 μM Pep1 **(B)** treatment was applied at around 540 s. **(D,E)** *Arabidopsis* seedlings were subjected to a 1 μM Pep1 treatment once at 0 s, and the solution was perfused by deionized water at 420 s. Then, a second 1 μM Pep1 **(D)**, or 1 μM flg22 **(E)** treatment was applied at around 540 s. Aequorin luminescence was recorded continuously through the treatments in the dark. **(C,F)** Quantification of [Ca^2+^]_i_ increases for 1 μM flg22 and 1 μM Pep1 treatment from experiments as in **(A)** to **(B)**, and **(D)** to **(E)**, respectively. Data for four independent experiments are shown (mean ± SD; *n* = 16; NS, not significant *P* > 0.05; ^∗∗∗^*P* < 0.001).

To further characterize the potential interaction between the different biotic stress stimuli-triggered [Ca^2+^]_i_ signaling, plants were treated either with the same or different stimulus. When the *Arabidopsis* seedlings were treated with 1 μM flg22, the level of [Ca^2+^]_i_ increased quickly to reach a peak, then decreased to the new resting level after 400 s (**Figure 2A**), as described in **Figure 1A**. A subtle increase in [Ca^2+^]_i_ could be detected in seedlings after they had been washed with deionized water at around 500 s (**Figure 2A**; green). Next, flg22 was added again, which resulted in a very minor increase in [Ca^2+^]_i_. After 800 s, it decayed to a level similar to the previous resting level. Compared with the first flg22 treatment, which led to a large [Ca^2+^]_i_ increase, the second flg22 treatment resulted in a [Ca^2+^]_i_ increase that was only a fraction of the size of the first [Ca^2+^]_i_ increase. This observation suggests that the flg22-activated Ca^2+^ permeable channel may be desensitized or adapted by some unknown signaling elements upstream. To test whether the desensitization or adaptation occurs, we can (after waiting for 3 h) detect a normal [Ca^2+^]_i_ increase in response to flg22. This result suggests that desensitization of the channel is likely to happen, which agrees with the results reported by Heese’s group (Smith et al., 2014). Subsequently, we analyzed whether the MAMP-activated Ca^2+^ permeable channel was affected by the initial MAMP treatment. The second flg22 treatment was replaced by a treatment with 1 μM Pep1 at 600 s (**Figure 2B**). Interestingly, the peak of [Ca^2+^]_i_ induced by 1 μM Pep1 was clearly greater than that of 1 μM flg22 (*P* < 0.001). After 900 s, the [Ca^2+^]_i_ decreased to a new basal level (**Figure 2B**). The lower inhibition of the Pep1-induced [Ca^2+^]_i_ increase compared with the increase induced by the initial flg22 treatment suggests that the initial high level of [Ca^2+^]_i_, which resulted from the flg22 treatment, inhibited flg22 to a greater extent than Pep1 (**Figures 2A,B**). By analogy, we used Pep1 as the first stimulus to treat the seedlings, and then analyzed the second treatment using flg22 or Pep1. When the second Pep1 was added to the Petri dish, following the first Pep1 treatment and water washing step at around 540 s the [Ca^2+^]_i_ level stabilized to a point similar to previous resting levels (**Figures 2D,E**). The column chart has also been used to clearly show the results, as in **Figures 2C,F**. However, when we used 1 μM flg22 to replace Pep1 at 600 s, the peak value was smaller but significantly higher than that induced by the second Pep1 treatment (**Figures 2D,E**). Similarly, our results suggested that the high [Ca^2+^]_i_ resulting from the initial Pep1 activation inhibited the MAMP-induced [Ca^2+^]_i_ to a greater degree than the PAMP-induced [Ca^2+^]_i_ (**Figure 2**).

### The Crosstalk between Biotic and Abiotic Stresses-Triggered [Ca^2+^]_i_ Increases

**FIGURE 3:**
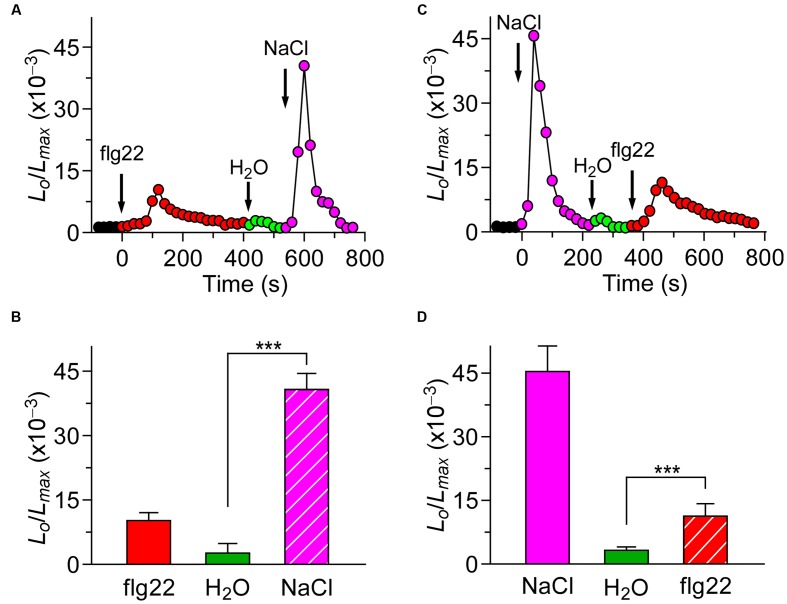
**flg22 and NaCl induced [Ca^2+^]_i_ increases do not influence each other.** **(A)** *Arabidopsis* seedlings were subjected to a 1 μM flg22 treatment once at 0 s, and the solution was perfused by deionized water at 420 s. Then, 200 mM NaCl treatment was applied at around 540 s. **(C)** *Arabidopsis* seedlings were subjected to a 200 mM NaCl treatment once at 0 s, and the solution was perfused by deionized water at 220 s. Then, 1 μM flg22 treatment was applied at around 340 s. Aequorin luminescence was recorded continuously through the treatments in the dark. **(B,D)** Quantification of [Ca^2+^]_i_ increases for 1 μM flg22 and 200 mM NaCl treatment from experiments as in **(A)** to **(C)**, respectively. Data for four independent experiments are shown (mean ± SD; *n* = 16; NS, not significant *P* > 0.05; ^∗∗∗^*P* < 0.001).

**FIGURE 4:**
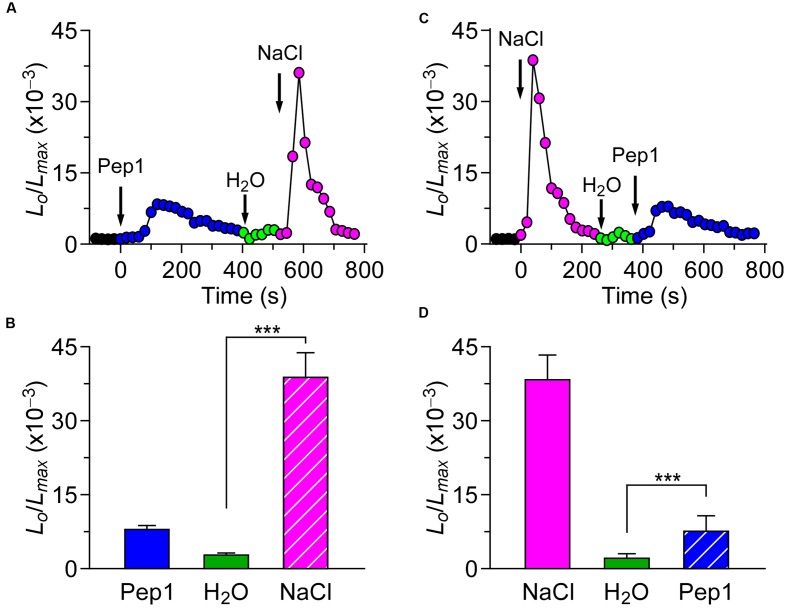
**Pep1 and NaCl induced [Ca2+]i increases do not influence each other.** **(A)** *Arabidopsis* seedlings were subjected to a 1 μM Pep1 treatment once at 0 s, and the solution was perfused by deionized water at 420 s. Then, 200 mM NaCl treatment was applied at around 540 s. **(C)** *Arabidopsis* seedlings were subjected to a 200 mM NaCl treatment once at 0 s, and the solution was perfused by deionized water at 220 s. Then, 1 μM Pep1 treatment was applied at around 340 s. Aequorin luminescence was recorded continuously through the treatments in the dark. **(B,D)** Quantification of [Ca^2+^]_i_ increases for 1 μM Pep1 and 200 mM NaCl treatment from experiments as in **(A)** to **(C)**, respectively. Data for four independent experiments are shown (mean ± SD; *n* = 16; NS, not significant *P* > 0.05; ^∗∗∗^*P* < 0.001).

Based on our study, we know that different biotic stresses may induce [Ca^2+^]_i_ increases via different channels. It is of great importance to further characterize the interaction between biotic and abiotic stress stimuli-triggered [Ca^2+^]_i_ signaling. For such a purpose, we treated the plants with both the biotic and abiotic stimulus. When the *Arabidopsis* seedlings were treated with 1 μM flg22, the level of [Ca^2+^]_i_ increased rapidly to reach a peak, and decreased to the new resting level after 400 s. A subtle increase in [Ca^2+^]_i_ could be detected in the seedlings after they were washed in deionized water at around 500 s (**Figure 3A**; green). Next, NaCl was added, which caused a sharp increase in [Ca^2+^]_i_ (**Figure 3A**). The [Ca^2+^]_i_ then decayed from 700 s to a level similar to previous resting level. Compared with the single NaCl treatment, which led to a large increase in [Ca^2+^]_i_ increase (**Figure 3C**), the NaCl treatment after the flg22 stimulus still resulted in an increase in [Ca^2+^]_i_ that was similar to a single NaCl-induced increase in [Ca^2+^]_i_. This observation suggests that there may be no interaction between the NaCl-activated Ca^2+^ permeable channel (NaC) and flg22-activated Ca^2+^ permeable channel. To verify this hypothesis, we treated the seedlings with 200 mM NaCl. The [Ca^2+^]_i_ increased quickly to reach a peak and then decreased to the new resting level after 150 s (**Figure 3C**). Next, we added 1 μM of flg22, which caused an increase in [Ca^2+^]_i_ similar to the effect with a single flg22 treatment. Subsequently, we used a DAMP elicitor Pep1 instead of flg22 to determine whether a DAMP-induced [Ca^2+^]_i_ increase can affect an NaC. To clearly show the results, column chart were used in **Figures 3B,D**. As expected, it appeared that there was no interaction between the NaC and Pep1-activated Ca^2+^ permeable channel (**Figures 4A–D**). Based on this study, our results suggest that abiotic and biotic stress stimuli-activated Ca^2+^ permeable channels may be completely independent of each other.

### The Additive Effect of flg22, Pep1, and NaCl on Triggering Increases in [Ca^2+^]_i_

**FIGURE 5:**
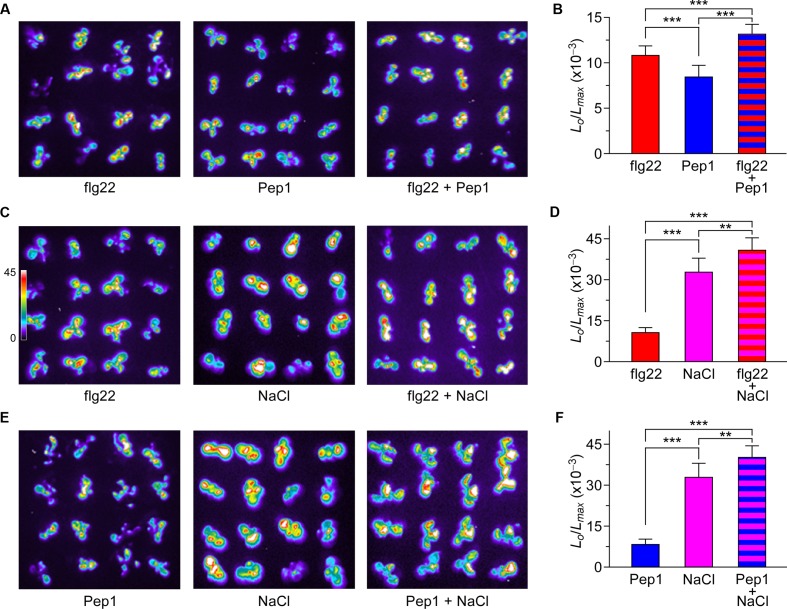
**Increases in [Ca^2+^]_i_ in response to flg22, Pep1, and NaCl individually or combined.** **(A)** Imaging of [Ca^2+^]_i_ increases in response to the treatments of 1 μM flg22, 1 μM Pep1, and together. **(C)** Imaging of [Ca^2+^]_i_ increases in response to the treatments of 1 μM flg22, 200 mM NaCl, and together. **(E)** Imaging of [Ca^2+^]_i_ increases in response to the treatments of 1 μM Pep1, 200 mM NaCl, and together in *Arabidopsis* seedlings expressing aequorin. All images were taken for 420 s, and [Ca^2+^]_i_ increases were analyzed by imaging bioluminescence and scaled by a pseudo-color bar. **(B,D,F)** Quantification of [Ca^2+^]_i_ increases from experiments as in **(A,C,E)**. Data for four independent experiments are shown (mean ± SD; *n* = 16; ^∗∗∗^*P* < 0.001; ^∗∗^0.001 < *P* < 0.01; NS, not significant *P* > 0.05).

To investigate thoroughly the relationship and interaction between [Ca^2+^]_i_ increases triggered by biotic and abiotic stresses, *Arabidopsis* seedlings were treated with 1 μM flg22, 1 μM Pep1, or 200 mM NaCl separately, or any two of these three elicitors. The [Ca^2+^]_i_ increases recorded after single treatments were consistent with the results described above. When plants were treated with 1 μM flg22 and 1 μM Pep1 together, the peaks of [Ca^2+^]_i_ were slightly larger than those induced by each individual stimulus (**Figure 5A**). However, when plants were treated with 200 mM NaCl together with 1 μM flg22 or 1 μM Pep1, the peaks of [Ca^2+^]_i_ were larger than those induced by each individual stimulus, showing an additive effect (**Figures 5C,E**). To further analyze the difference in [Ca^2+^]_i_ increases in response to both biotic and abiotic stresses, we calculate the [Ca^2+^]_i_ to clearly illustrate the data. As shown in **Figure 5B**, compared with individual treatment with Pep1, plants treated with flg22 and Pep1 together show only a slight increase in [Ca^2+^]_i_. However, compared with individual treatment with NaCl, plants treated with flg22 and NaCl together, or Pep1 and NaCl together, show an increase in [Ca^2+^]_i_ that is almost equal to that shown with the combined treatment (**Figures 5D,F**). These results suggest that the flg22-induced and Pep1-induced [Ca^2+^]_i_ increases may operate through similar Ca^2+^ permeable channels, though biotic stress-induced [Ca^2+^]_i_ increases may occur as independent events. In other words, biotic and abiotic stresses may activate different Ca^2+^ permeable channels.

### Calcium Signaling Induced by Biotic and Abiotic Stresses Are Independent in Terms of Spatial and Temporal Patterning

**FIGURE 6:**
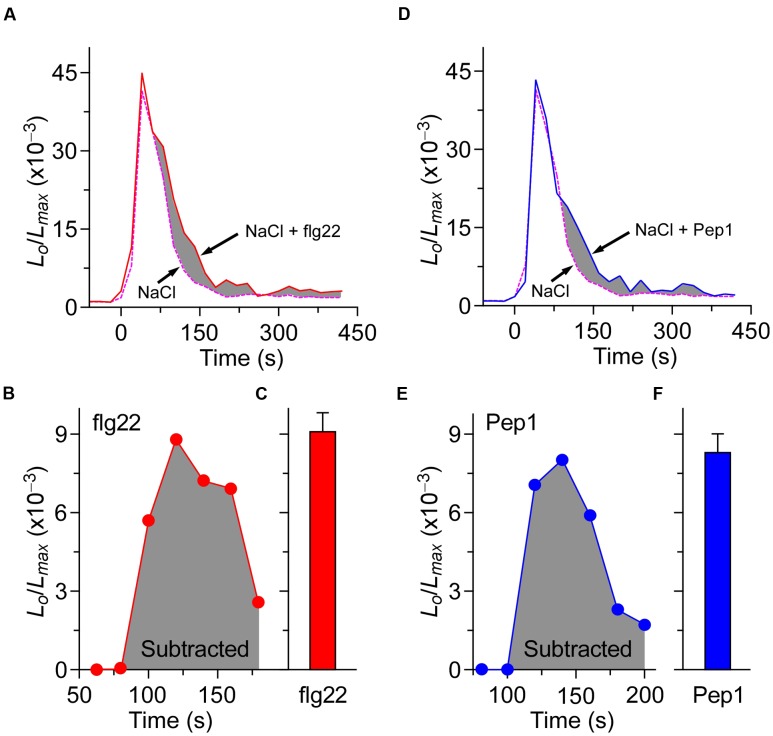
**The processes of [Ca^2+^]_i_ increases induced by biotic and abiotic stresses are independent.** **(A)** Time courses of increase in [Ca^2+^]_i_ induced by 200 mM NaCl (dashed line) and 200 mM NaCl together with 1 μM flg22 (full line). **(D)** Time courses of increase in [Ca^2+^]_i_ induced by 200 mM NaCl (dashed line) and 200 mM NaCl together with 1 μM Pep1 (full line). Seedlings grown for 7 days were treated at time 0, and aequorin images were taken every 20 s. **(B,C)** Increases in [Ca^2+^]_i_ induced by 1 μM flg22 that calculated from **(A)**. **(E,F)** Increases in [Ca^2+^]_i_ induced by 1 μM Pep1 that calculated from **(D)**. Similar results were seen in four independent experiments using 256 seedlings.

The co-treatment of biotic and abiotic stresses triggers an additive effect on the increase of [Ca^2+^]_i_. Treated either by flg22 with NaCl or Pep1 with NaCl, the increase of [Ca^2+^]_i_ could indicate that biotic and abiotic stresses activate different Ca^2+^ permeable channels. While it is not clear whether the treatment of NaCl will affect the calcium signal process and peak value, which are caused by the flg22/Pep1 treatment. To answer this question, in our experiments, seedlings were treated either by 1 μM flg22 along with 200 mM NaCl or 1 μM Pep1 along with 200 mM NaCl. The results are shown in **Figure 6**. The shaded area in **Figures 6A,D** illustrates the difference between the co-treated plants and NaCl-only treated plants. The subtracted shaded area reveals that the dynamic process and peak value of [Ca^2+^]_i_ achieved by the treatment of using both flg22 and Pep1 are similar to those delivered by using the flg22 or Pep1 alone (**Figures 6B,C,E,F**). These results indicate that biotic and abiotic stresses induce the increase of [Ca^2+^]_i_ through different Ca^2+^ permeable channels. Such results are consistent with our prediction.

## Discussion

Calcium is the most important secondary messenger and plays an essential role in signal transduction throughout the lives of both animals and plants (Berridge et al., 2003; Clapham, 2007; Ward et al., 2009; Ranf et al., 2011). Changes in [Ca^2+^]_i_ in response to various abiotic and biotic stresses (including pathogen elicitors, salt stress, drought stresses, oxidative stress, and high and low temperatures) in plants have been a topic of much interest over the past two decades (McAinsh and Pittman, 2009; Dodd et al., 2010; Ma and Berkowitz, 2011; Yuan et al., 2014). Specific stimuli can trigger unique temporal and spatial patterns of [Ca^2+^]_i_ known as “[Ca^2+^]_i_ signatures” (Tang et al., 2007; Spalding and Harper, 2011). The [Ca^2+^]_i_ signature encodes information from the environmental stimulus which will be decoded subsequently by intracellular Ca^2+^ sensors, such as calcium-dependent protein kinases, calmodulins, and calcineurin B-like proteins, leading to the activation of downstream events (Galon et al., 2008; Jeworutzki et al., 2010; Stael et al., 2012; Steinhorst and Kudla, 2013; Seybold et al., 2014). Basal [Ca^2+^]_i_ is controlled below the extracellular Ca^2+^ concentration at a concentration round 10,000-fold (Berridge et al., 2003; Clapham, 2007; Swanson et al., 2011). Generally, in response to environmental stimuli, Ca^2+^ channels in the plasma membrane and/or endomembranes can be activated and lead to the increases of [Ca^2+^]_i_ (Hetherington and Brownlee, 2004; Ward et al., 2009; Ranf et al., 2011). Biotic and abiotic stress-induced [Ca^2+^]_i_ increases have traditionally been considered to be involved in the procedure of perceiving the stress signaling, though the molecular nature of this process is poorly understood (Luan et al., 2009; Ranf et al., 2011). One recent study has shown that OSCA1 is a plasma membrane protein, which can be used to form hyperosmolality-gated calcium-permeable channels. This study reveals that OSCA1 could be served as an osmosensor. The OSCA1 represents a channel responsible for the increases of [Ca^2+^]_i_ induced by a stimulus in plants, leading to a new avenue to study Ca^2+^ processes in relation to other stimuli (Yuan et al., 2014). Observing the lag phases and [Ca^2+^]_i_ amplitudes in plant’s early response, we found that [Ca^2+^]_i_ increases induced by abiotic stresses are similar. Based on the above results we speculate that abiotic stress-induced [Ca^2+^]_i_ signaling are mediated via a sensory channel. Thus, in contrast to abiotic stresses, the biotic stress-induced [Ca^2+^]_i_ oscillation curve presents different lag phases and [Ca^2+^]_i_ amplitudes. This demonstrates that both abiotic stress-induced and biotic stress-induced [Ca^2+^]_i_ increases may utilize entirely different channels.

It is well known that recognition of PAMPs or DAMPs by PRRs leads to a first line of inducible defenses that restrict microbial propagation in multicellular organisms (Boller and Felix, 2009; Gupta et al., 2011; Kawai and Akira, 2011; Segonzac and Zipfel, 2011; Zipfel, 2014; Bigeard et al., 2015; Yamada et al., 2016). Although it was reported many years ago that a rapid change in the cytosolic Ca^2+^ concentration ([Ca^2+^]_i_) and concomitant membrane depolarization follows MAMP/DAMP perception, the interaction and interrelationship between many early MAMP/DAMP signaling components in *Arabidopsis* are not well understood. The lessening of the [Ca^2+^]_i_ increases induced by both MAMPs and DAMPs observed in this study (**Figures 1D,E**) suggests that the feedback inhibitory mechanism could inactivate the stimulus-activated Ca^2+^ permeable channels. Briefly speaking, elevated [Ca^2+^]_i_ will inhibit the ion channels in plants. We speculate that this phenomenon may be similar to the depolarization process of receptor ion channels typically seen in animals (Traynelis et al., 2010). One particular study reported that such receptor desensitization also occurs in plants (Smith et al., 2014). However, we did not observe any [Ca^2+^]_i_ at 420 s after flg22 or Pep1 treatment (**Figures 1D,E**).

**FIGURE 7:**
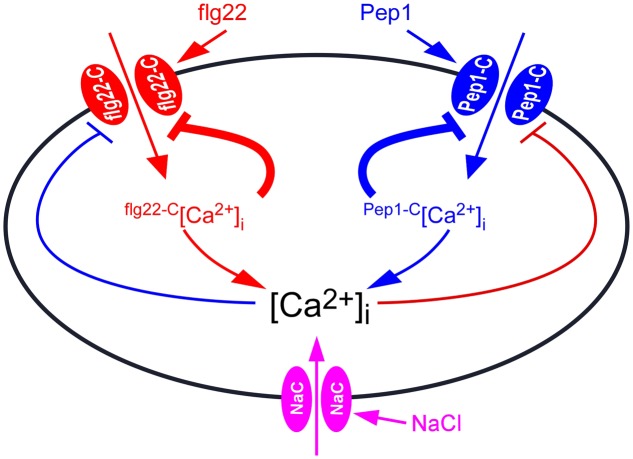
**Model for the interaction between biotic and abiotic stresses-induced [Ca^2+^]_i_ increases.** Ca^2+^ channel activated by flg22 (flg22-C) results in localized [Ca^2+^]_i_ increases, called flg22-C-related [Ca^2+^]_i_ microdomain (flg22-C[Ca^2+^]_i_). The flg22-C[Ca^2+^]_i_ feedback inhibits the activity of flg22-C. Pep1-C, a Ca^2+^ channel activated by hydrogen peroxide, leads to localized [Ca^2+^]_i_ increases, called Pep1-C [Ca^2+^]_i_ microdomain. Pep1-C [Ca^2+^]_i_ also feedback inhibits Pep1-C activity. The [Ca^2+^]_i_ microdomain-mediated inhibition of Ca^2+^ channels is the major feedback inhibitory pathways (thick lines). In addition, both flg22-C[Ca^2+^]_i_ and Pep1-C[Ca^2+^]_i_ might contribute to a NaCl-activated Ca^2+^ permeable channels (NaC), which further inhibits both flg22-C and Pep1-C, serving as biotic and abiotic stresses feedback inhibitory pathways (thin lines). [Ca^2+^]_i_ is reset to the resting level by plasma membrane Ca^2+^ pumps.

flg22 and Pep1 induced slightly increases of [Ca^2+^]_i_ than using either flg22 or Pep1 alone (**Figure 5**), indicating that flg22 and Pep1 may, in part, share Ca^2+^ permeable channels flg22-C and Pep1-C (**Figure 7**). The flg22-C and Pep1-C are likely regulated by feedback inhibition (**Figure 7**), considering their desensitization seen in this study (**Figures 2A,D**) as well as in previous reports. We demonstrated that repetitive flg22 treatments failed to trigger repetitive [Ca^2+^]_i_ increases (**Figures 2A,C**). This indicates that the flg22-C cannot be activated repetitively within a short period of time—that is, flg22-C is possibly desensitized. We can therefore deduce that a feedback inhibition may be involved in the desensitization process (**Figure 7**). Upon flg22 treatment, the flg22-C opens, leading to a localized increase in [Ca^2+^]_i_, flg22-C [Ca^2+^]_i_ microdomain/puff. The flg22-C [Ca^2+^]_i_, in turn, signals the channel to close, which prevents further [Ca^2+^]_i_ increases and allows the basal [Ca^2+^]_i_ to be reset via Ca^2+^ pumps. Such feedback inhibition avoids any excessive increase in [Ca^2+^]_i_, which could be highly deleterious to plant cells. The same phenomenon was also observed with the activation of Pep1-C (**Figure 7**). Clearly, the most significant effect was observed after the initial treatment by flg22-C or Pep1-C, when the rate of [Ca^2+^]_i_ increases induced by both flg22-C and Pep1-C decreased (**Figure 2**). It is most probably that localized flg22-C [Ca^2+^]_i_ and Pep1-C [Ca^2+^]_i_ merge to form a obviously global [Ca^2+^]_i_, the feedback of which then inhibits both flg22-C and Pep1-C (**Figure 5**). In contrast, flg22 or Pep1, together with NaCl, induced greater increases in [Ca^2+^]_i_ than using flg22 or Pep1 alone, leading us to conclude that there is no interrelationship between the NaCs. It should be noted that our study does not prove that flg22-C, Pep1-C, and NaC are localized in discrete and different microdomains, instead illustrates that flg22-C, Pep1-C, and NaC may differ and interact via [Ca^2+^]_i_ microdomains.

To some extent, PAMP- and DAMP-induced [Ca^2+^]_i_ increases differ, but they all belong to the same pattern. In plants, biotic stresses that include PAMP- and DAMP-induced [Ca^2+^]_i_ increases are similar in spatial and temporal patterning. Thus, we treated plants with flg22 together with Pep1, and found that the [Ca^2+^]_i_ peaks were slightly larger than those induced by each stimulus alone, showing an enhanced signaling mechanism (**Figure 5**). This is similar to NaCl- and H_2_O_2_-induced [Ca^2+^]_i_ increases (Jiang et al., 2013), suggesting that PAMPs and DAMPs may partly activate the same Ca^2+^ permeable channel. When plants were treated with NaCl together with flg22 or Pep1, the [Ca^2+^]_i_ peaks were larger than those induced by each individual stimulus, showing an additive effect (**Figures 5C,E**). These results suggest that biotic and abiotic stresses may activate different Ca^2+^ permeable channels. In **Figure 6**, we present the calcium signal oscillation curve within the same image. We noticed that the calcium signal induced by biotic and abiotic stresses was independent in terms of spatial and temporal patterning. This study further demonstrates that early signaling is relatively independent to biotic and abiotic stresses.

Plants resist biotic and abiotic stresses by triggering two different sets of calcium signaling pathways. This is of great significance to the plant’s survival strategies. It will be important for future research to analyze the pharmacological properties of these putative Ca^2+^ permeable channels activated by flg22. Clearly, identifying these channels or sensors is extremely important to study the plant stresses resistance. Additionally, how flg22-C and Pep1-C interact thus contributing to the development of [Ca^2+^]_i_ signatures as well as other downstream events could be analyzed further once their molecular nature being revealed.

## Author Contributions

SZ, Z-MP, and Z-HJ conceived and designed the experiments. X-QC, SZ, Y-YY, and YY performed the experiments. SZ, X-QC, and Z-MP analyzed the data. L-PK contributed reagents/materials/analysis tools. SZ wrote the manuscript.

## Conflict of Interest Statement

The authors declare that the research was conducted in the absence of any commercial or financial relationships that could be construed as a potential conflict of interest.

## Footnotes

1 http://www.chinapeptides.net
